# Breastfeeding difficulties in the first 6 weeks postpartum among mothers with chronic conditions: a latent class analysis

**DOI:** 10.1186/s12884-023-05407-w

**Published:** 2023-02-02

**Authors:** Natalie V. Scime, Amy Metcalfe, Alberto Nettel-Aguirre, Kara Nerenberg, Cynthia H. Seow, Suzanne C. Tough, Kathleen H. Chaput

**Affiliations:** 1 Department of Community Health Sciences, University of Calgary, Calgary, AB Canada; 2 Department of Obstetrics and Gynecology, University of Calgary, Calgary, AB Canada; 3 Department of Medicine, University of Calgary, Calgary, AB Canada; 4 Centre For Health and Social Analytics, School of Mathematics and Statistics, National Institute for Applied Statistical Research, University of Wollongong, Wollongong, NSW Australia; 5 Department of Pediatrics, University of Calgary, Calgary, AB Canada; 6 Division of General Internal Medicine, Department of Medicine, University of Calgary, Calgary, AB Canada; 7 Division of Gastroenterology & Hepatology, Department of Medicine, University of Calgary, Calgary, AB Canada

**Keywords:** Breast feeding, Chronic disease, Postpartum period, Prospective studies

## Abstract

**Background:**

Breastfeeding difficulties frequently exacerbate one another and are common reasons for curtailed breastfeeding. Women with chronic conditions are at high risk of early breastfeeding cessation, yet limited evidence exists on the breastfeeding difficulties that co-occur in these mothers. The objective of this study was to explore clusters of breastfeeding difficulties experienced up to 6 weeks postpartum among mothers with chronic conditions and to examine associations between chronic condition types and breastfeeding difficulty clusters.

**Methods:**

We analyzed 348 mothers with chronic conditions enrolled in a prospective, community-based pregnancy cohort study from Alberta, Canada. Data were collected through self-report questionnaires. We used latent class analysis to identify clusters of early breastfeeding difficulties and multinomial logistic regression to examine whether types of chronic conditions were associated with these clusters, adjusting for maternal and obstetric factors.

**Results:**

We identified three clusters of breastfeeding difficulties. The “physiologically expected” cluster (51.1% of women) was characterized by leaking breasts and engorgement (reference outcome group); the “low milk production” cluster (15.4%) was discerned by low milk supply and infant weight concerns; and the “ineffective latch” cluster (33.5%) involved latch problems, sore nipples, and difficulty with positioning. Endocrine (adjusted relative risk ratio [RRR] 2.34, 95% CI 1.10–5.00), cardiovascular (adjusted RRR 2.75, 95% CI 1.01–7.81), and gastrointestinal (adjusted RRR 2.51, 95% CI 1.11–5.69) conditions were associated with the low milk production cluster, and gastrointestinal (adjusted RRR 2.44, 95% CI 1.25–4.77) conditions were additionally associated with the ineffective latch cluster.

**Conclusion:**

Half of women with chronic conditions experienced clusters of breastfeeding difficulties corresponding either to low milk production or to ineffective latch in the first 6 weeks postpartum. Associations with chronic condition types suggest that connections between lactation physiology and disease pathophysiology should be considered when providing breastfeeding support.

**Supplementary Information:**

The online version contains supplementary material available at 10.1186/s12884-023-05407-w.

## Background

The advantages of breastfeeding over formula feeding for maternal and child health are well established, including lowered risk of infection, obesity, and asthma in children and reduced risk of cardiovascular morbidity and breast and ovarian cancers for mothers [1–5]. Breastfeeding is recommended as the primary source of infant nutrition until 6 months when complementary foods are introduced, and sustained for longer–up to 2 years and beyond–according to maternal preference [6]. Yet a substantial proportion of women discontinue breastfeeding earlier than is recommended or planned [7, 8]. Difficulties with the mechanics of breastfeeding and physiology of lactation are the most commonly cited reasons for early cessation [8–10]. Women who have breastfeeding difficulties often report intense feelings of inadequacy, failure, and powerlessness and face higher risk for postpartum depression [11–14], particularly in light of inadequate lactation support [15]. Public health emphasis on breastfeeding promotion should therefore be matched with high-quality care to prevent and address breastfeeding difficulties [16], taking into account that difficulties frequently cluster together and exacerbate one another [17].

Increasingly, evidence has shown that mothers with pre-existing physical health conditions are at high risk of early breastfeeding cessation compared to the general maternal population [7, 18–20]. Evidence on the clusters of breastfeeding difficulties in women with chronic conditions that may underpin this disparity is scant [21]. Moreover, our understanding of whether the type of chronic condition, each with its own set of pathological and clinical features and management, impacts lactation through breastfeeding difficulties is limited. For example, Berg et al. reported that mothers with type 1 diabetes were more likely to report low milk supply than mothers without diabetes at 2 months postpartum. Hormonal aberrations from diabetes are thought to reduce milk production; [22] this may extend to other endocrine conditions such as thyroid disorders [23, 24], though few epidemiologic studies have investigated this. Qualitative studies on mothers with musculoskeletal conditions have detailed distinct challenges with breastfeeding positioning and latch due to pain and mobility limitations [25–29]. We therefore sought to explore clusters of breastfeeding difficulties experienced by mothers with a wide range of chronic conditions up to 6 weeks postpartum, and to examine potential associations between chronic condition types and breastfeeding difficulty clusters.

## Methods

### Study design

We conducted a prospective, community-based pregnancy cohort study of women with pre-existing physical health conditions in Alberta, Canada called the Motherhood and Chronic Illness (MaCI) Study. Our overarching aim for the MaCI Study was to explore factors associated with breastfeeding intentions, difficulties, support experiences, and outcomes among mothers with chronic conditions; we were specifically focused on exploring factors unique to maternal chronic conditions (e.g., condition types), hence the simultaneous inclusion of a healthy comparator group was inapplicable to our focus. The Conjoint Health Research Ethics Board at the University of Calgary approved this study (REB19-0443), and all participants provided informed consent upon enrollment.

### Study sample

**Fig. 1:**
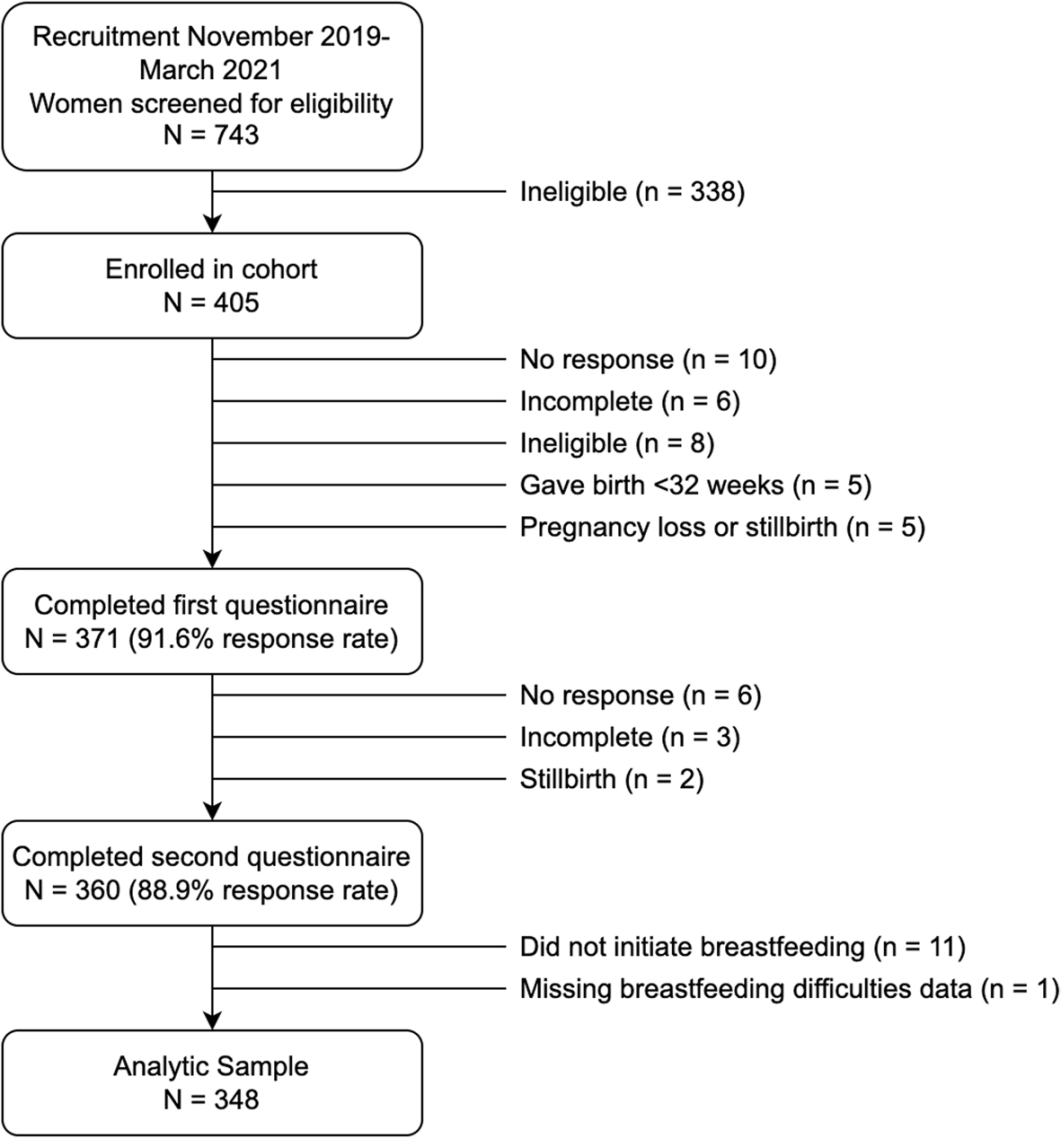
Flowchart of cohort recruitment and selection of analytic sample

Women were eligible if they were living with at least one chronic physical health condition (defined using the Agency for Healthcare Research and Quality Chronic Condition Indicator) [30], carrying a singleton pregnancy less than 32 weeks gestation, aged 18 or older, planning to try breastfeeding or expressing breast milk after birth, able to complete online questionnaires in English, and residing in Alberta, Canada. We recruited participants through obstetric clinics, social media advertisements, targeted mailings through the province’s health authority, and word of mouth from November 2019 to March 2021. We screened a total of 743 women, of whom 405 met the eligibility criteria and were enrolled in the study (Fig. 1).

### Data collection

Participants completed a total of three online questionnaires at 32 weeks gestation, 6 weeks postpartum, and 6 months postpartum that together collected information on reproductive history, obstetrical events, chronic illnesses and medications, health behaviours (e.g., sleep, substance use), mental health, infant feeding, and social determinants of health. We used data from the first two questionnaires for this analysis to maintain focus on the early postpartum period when risk of breastfeeding difficulties and cessation are highest [31].

### Measurement of breastfeeding difficulties

Breastfeeding difficulties known to commonly occur in the early postpartum, based on existing literature [9, 32, 33], were measured at 6 weeks following delivery: feeling tired/fatigued, sore nipples, cracked nipples, swollen/engorged breasts, leaking breasts, baby having trouble latching on, baby too sleepy during feeds, baby too fussy during feeds, baby feeding too frequently, not enough milk, difficulty positioning baby during feeds, and feeling worried about baby’s weight gain or loss. Participants were asked to indicate the perceived severity of each difficulty using a 4-point Likert scale of not at all, mild, moderate, or severe, considering their experiences from birth up to the time of the questionnaire [34]. While mild breastfeeding difficulties are highly prevalent in the early postpartum period (upwards of 90% in our sample) and tend to resolve, moderate and severe difficulties are generally more persistent, distressing, and obstructive to breastfeeding efforts [12, 13, 35, 36]. We therefore developed a binary variable for moderate-to-severe presence of each difficulty (i.e., collapsing not at all/mild and moderate/severe).

### Measurement of chronic condition types

Self-reported chronic conditions present in the cohort were classified using chapters in the International Classification of Diseases (ICD) 10^th^ Revision, in which medical conditions are grouped based on the affected body system, pathology, classical symptoms, and/or medical specialty responsible for care. Given that some women reported more than one condition, types were measured with a binary indicator for each ICD chapter. Chronic condition types (ICD chapter; title) included: hematologic (D; blood and blood-forming organs), endocrine (E; endocrine, nutritional, and metabolic), neurological (G; nervous system), cardiovascular (I; circulatory system), respiratory (J; respiratory system), gastrointestinal (K; digestive system), dermatologic (L; skin and subcutaneous tissue), musculoskeletal (M; musculoskeletal system and connective tissue), genitourinary (N; genitourinary system), and congenital (Q; congenital malformations, deformations, and chromosomal abnormalities). Owing to low prevalence of hematologic, dermatologic, and congenital conditions (each < 5%), indicators for these types were excluded from multivariable analysis; women with these conditions were not excluded, but rather contributed data to the remaining indicators based on additional morbidities present.

### Statistical analysis

First, we used latent class analysis (LCA) to explore clusters of breastfeeding difficulties. LCA is a statistical method that helps identify unobserved (latent) subgroups of individuals in a population based on response patterns to a set of observed (measured) variables [37]. LCA is a person-centred approach in that it focuses on identifying groups of individuals who share similar within-person characteristics, contrasting with the variable-centred approach which focuses on associations among variables [38]. All binary variables for moderate-to-severe (versus none-to-mild) breastfeeding difficulties were included in the analysis. We fit LCA models with 1 through 4 latent classes and jointly considered model fit indices (Akaike information criterion [AIC], Bayesian information criteria [BIC], sample size-adjusted BIC [SABIC], and log likelihood), model parsimony, and clinical utility of the groupings in selecting the final number of latent classes, herein termed clusters. Lower values indicate better fit for the AIC, BIC, and SABIC, while higher values indicate better fit for the log likelihood [39]. Once the final model was selected, we estimated each participant’s probability of belonging to each cluster and assigned group membership using the cluster with the highest probability.

Next, we used multinomial logistic regression to examine whether different types of chronic conditions were associated with breastfeeding difficulty clusters. Multinomial logistic regression yields relative risk ratios (RRR) and 95% confidence intervals (CI). All chronic condition type indicators were modelled simultaneously (i.e., one model was constructed with all indicators); the referent group for each indicator was women who did not report living with that specific type of chronic condition (but reported other chronic conditions, as per the study eligibility criteria). To address potential confounding, models were adjusted for the following covariates based on prior evidence: maternal age (years), pre-pregnancy body mass index (BMI; kg/m^2^), prenatal depression (score on the Edinburgh Postnatal Depression Scale [EPDS]) [40, 41], prenatal anxiety (score on the 6-item short-form Spielberger State-Trait Anxiety Inventory [STAI]) [42, 43], maternal education (post-secondary degree versus some post-secondary education or less), mode of delivery (vaginal versus Cesarean section), and obstetrical complications (composite binary variable for one or more of: gestational hypertension, preeclampsia, gestational diabetes, placental disorder, postpartum hemorrhage, or preterm birth < 37 weeks); owing to low prevalence (< 5%) in this sample, prenatal tobacco/nicotine use was not included in the models to maintain sufficient precision of point estimates. Missing covariate data were minimal (3%) and handled through complete case analysis. All analyses were performed in Stata MP version 17.

## Results

Of the 405 participants enrolled, 371 completed the 32-week pregnancy questionnaire (91.6% response rate) and 360 completed the 6-week postpartum questionnaire (88.9% response rate). From the 360 women who responded to both questionnaires, we excluded 11 women who did not initiate breastfeeding and 1 woman who did not provide complete data on breastfeeding difficulties, resulting in a sample size of 348 for this analysis (Fig. 1).

Table 1 describes characteristics of the MaCI Study sample used for this analysis. Participants were predominantly White, nulliparous or primiparous, reported a household income above the LICO for their area of residence, and held a post-secondary degree. Mean maternal age was 31.7 years and mean pre-pregnancy BMI was 27.4 kg/m^2^, which falls in the overweight range. Sample demographic characteristics were comparable to that of the baseline MaCI cohort and Alberta maternal population in recent years; however, the MaCI sample slightly under-represented mothers who were younger than 24 years of age, self-identified as BIPOC, did not hold a post-secondary degree, or were multiparous (Supplementary Table 1).

**Table 1:** Sample characteristics (*N* = 348)

	n	%
Sociodemographic		
Age in years, mean ± SD	31.7 ± 4.1	
Race/ethnicity		
White	271	78.1
Black, Indigenous, or Person of Colour	76	21.9
Household income below the LICO	72	20.7
Education		
Post-secondary degree	281	80.7
Less than post-secondary degree	67	19.3
Physical and Mental Health		
Pre-pregnancy body mass index in kg/m^2^, mean ± SD	27.4 ± 7.1	
Depressive symptoms: EPDS		
Score, mean ± SD	9.3 ± 5.2	
Score of ≥ 10	157	45.1
Anxiety symptoms: STAI		
Score, mean ± SD	38.6 ± 12.5	
Score of ≥ 40	166	47.7
Obstetrical		
Parity		
0	167	48.1
1	137	39.5
2 +	43	12.4
Mode of delivery		
Vaginal	220	63.2
Cesarean	128	36.8
Obstetrical complications	126	36.2
Chronic condition(s)		
Hematologic (e.g., anemia)	15	4.3
Endocrine (e.g., diabetes)	126	36.2
Neurological (e.g., multiple sclerosis)	46	13.2
Cardiovascular (e.g., hypertension)	32	9.2
Respiratory (e.g., asthma)	48	13.8
Gastrointestinal (e.g., Crohn’s disease)	76	21.8
Dermatologic (e.g., psoriasis)	12	3.4
Musculoskeletal (e.g., arthritis)	85	24.4
Genitourinary (e.g., endometriosis)	33	9.5
Congenital (e.g., Ehlers–Danlos syndrome)	15	4.3

*SD* Standard deviation, *EPDS* Edinburgh Postnatal Depression Scale, *STAI* Spielberger State-Trait Anxiety Inventory, *LICO* Low-income cut-off threshold (based on postal code)

Within the sample, prenatal mental health symptoms were slightly elevated; on average, women reported a score of 9.3 on the EPDS (a cut-off of 10 indicates elevated depressive symptoms) and 38.6 on the STAI (a cut-off of 40 indicates elevated anxiety symptoms). Nearly two thirds of women delivered vaginally and one third experienced obstetrical complications. Endocrine conditions were the most prevalent condition type, reported by 36.2% of women, followed by musculoskeletal (24.4%), gastrointestinal (21.8%), and neurological (13.2%) and respiratory (13.8%) conditions. Maternal fatigue (68.4%), leaking (44.0%) and engorged (35.9%) breasts, sore nipples (43.1%), low milk supply (32.5%), and latch problems (30.7%) were the most commonly reported breastfeeding difficulties rated as moderate to severe in the sample (Supplementary Table 2).

Model fit indices indicated that the 3- or 4-cluster model fit the data best, as evidenced by the lower values for the AIC, BIC, and SABIC and larger values for the log likelihood (Supplementary Table 3). Additional consideration of model parsimony and clinical utility of the clusters led us to select the 3-cluster model as the final model.

**Fig. 2:**
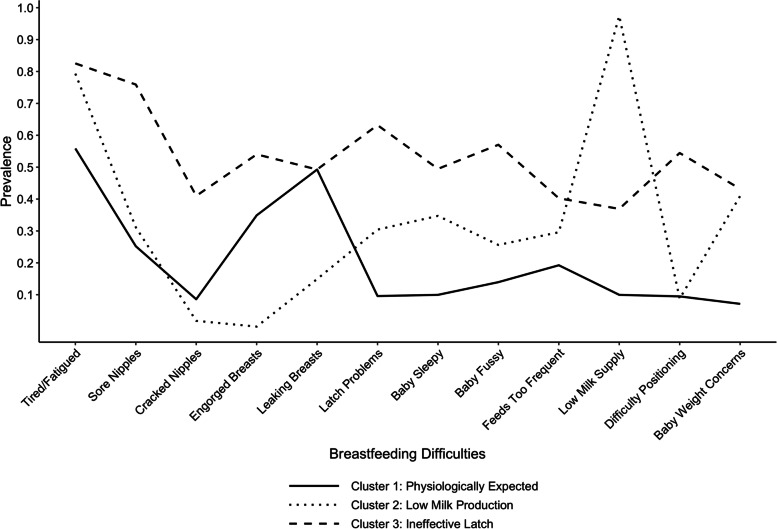
Breastfeeding difficulty clusters identified by latent class analysis. Line connections between points are not indicative of trend, as ordering of nominal categories along the X-axis is arbitrary; rather, lines are used to visually discern the patterns of prevalence of breastfeeding difficulties within each cluster

Figure 2 depicts the prevalence of each moderate-to-severe breastfeeding difficulty (compared to none-to-mild) for each of the three clusters. Cluster 1 was labelled “physiologically expected,” characterized by modest prevalence of leaking breasts (49.3%), engorgement (34.9%), and maternal fatigue (55.8%) and low prevalence of the remaining difficulties (not exceeding 20%). Cluster 2 was labelled “low milk production,” characterized by high prevalence of low milk supply (97.4%) and maternal fatigue (79.3%) and modest prevalence of concerns about infant weight gain (50.0%). Cluster 3 was labelled “ineffective latch,” characterized by high prevalence of sore nipples (75.9%), latch problems (63.2%), and maternal fatigue (82.5%) and modest prevalence of the remaining difficulties (35–55%). Most participants were assigned to the physiologically expected cluster (51.1%), followed by the ineffective latch (33.5%) and low milk production (15.4%) clusters. The distribution of breastfeeding difficulty clusters differed by method of breast milk feeding between birth and 6 weeks postpartum (Supplementary Table 4). The physiologically expected cluster was most prevalent in participants who fed only from the breast (69.3%); the low milk production cluster was most prevalent in participants who fed only expressed breast milk (38.1%); and the physiologically expected cluster was most prevalent (45.6%), followed by the ineffective latch cluster (37.7%), in participants who fed both at the breast and expressed milk.

Table 2 displays the results of multinomial logistic regression estimating the association between chronic condition types and breastfeeding difficulty clusters, using the physiologically expected cluster as the reference outcome group. Endocrine (adjusted RRR 2.34, 95% CI 1.10–5.00), cardiovascular (adjusted RRR 2.75, 95% CI 1.01–7.81), and gastrointestinal (adjusted RRR 2.51, 95% CI 1.11–5.69) conditions were associated with higher risk of belonging to the “low milk production” cluster. Gastrointestinal conditions were associated with higher risk of belonging to the “ineffective latch” cluster (adjusted RRR 2.44, 95% CI 1.25–4.77).

**Table 2:** Association between chronic condition types and breastfeeding difficulty clusters

	Relative Risk Ratio (95% CI)			
	Crude		Adjusted	
Low milk production vs. Physiologically expected (reference				
Endocrine	2.82	(1.38–5.80)	2.34	(1.10–5.00)
Neurological	0.90	(0.33–2.45)	0.89	(0.32–2.51)
Cardiovascular	3.53	(1.37–9.12)	2.75	(1.01–7.81)
Respiratory	1.02	(0.34–3.06)	0.95	(0.30–2.99)
Gastrointestinal	2.75	(1.23–6.12)	2.51	(1.11–5.69)
Musculoskeletal	0.97	(0.44–2.14)	0.80	(0.35–1.85)
Genitourinary	0.75	(0.26–2.18)	0.71	(0.22–2.24)
Ineffective latch vs. Physiologically expected (reference)				
Endocrine	1.66	(0.92–3.00)	1.75	(0.91–3.36)
Neurological	1.09	(0.52–2.31)	1.03	(0.45–2.33)
Cardiovascular	1.49	(0.59–3.73)	1.52	(0.54–4.28)
Respiratory	1.88	(0.91–3.90)	1.98	(0.89–4.40)
Gastrointestinal	2.40	(1.27–4.53)	2.44	(1.25–4.77)
Musculoskeletal	1.22	(0.67–2.22)	1.17	(0.62–2.23)
Genitourinary	0.51	(0.21–1.27)	0.37	(0.13–1.02)

Adjusted model controlled for maternal age, pre-pregnancy body mass index, prenatal depressive score, prenatal anxiety score, maternal education, mode of delivery, and obstetrical complications

*CI* Confidence interval

## Discussion

In this community-based cohort study of 348 postpartum mothers with chronic conditions, we identified three clusters of breastfeeding difficulties in the first 6 weeks postpartum. Half of mothers (49.3%) experienced physiologically expected difficulties of fatigue, leaking breasts, and engorgement. One third (33.5%) experienced ineffective latch, where latch problems co-occurred with several other difficulties including nipple pain. One in six (15.4%) experienced low milk production, where low milk supply co-occurred with concerns about infant weight gain.

Existing research has rarely accounted for the interrelatedness of breastfeeding difficulties, often studying them individually or as a composite [32, 44]. Exploratory factor analysis has been used to group similar types of breastfeeding difficulties [9, 14], but in the context of women’s underlying reasons for breastfeeding cessation. This approach overlooks women who are still breastfeeding despite potential difficulties or whose difficulties have resolved. Building on existing work, we used LCA to identify that most mothers with chronic conditions experienced co-occurring fatigue and breast overfullness in the first 6 weeks postpartum, which corresponds to the physiologically expected trajectory of breastfeeding establishment. Milk supply is upregulated to meet rapid increases in infant intake requirements, which mothers may experience as breast engorgement and leaking, and consolidated sleep is interrupted by frequent feeds and newborn care [45].

Sore nipples are experienced by up to 80% of breastfeeding mothers [44, 46]; while etiology is multifactorial, the most frequent cause is improper infant latch at the breast [47]. We identified an ineffective latch cluster which captured this causal link as well as co-occurring challenges with infant sleepiness, fussiness, and weight gain concerns, difficulty positioning the infant at the breast, and breast engorgement. Improper latch can lead to suboptimal draining of milk from the breasts during feeds, which initially presents as engorgement but will downregulate milk supply when sustained over time [48]. Moreover, limited transfer of milk to the infant can result in increased hunger cues and slower weight gain [48]. Our analysis of chronic condition types revealed that gastrointestinal conditions were associated with the ineffective latch cluster. Physical sensations of infant oral grasping and suckling on nipple tissue may be more painful for these women given that hypersensitivity to pain is a feature of several gastrointestinal conditions [49, 50]. Pain and discomfort in the abdominal area may also interfere with comfortable positioning of the infant to avoid cross-body breastfeeding positions.

Perceived low milk supply in the absence of self-reported latch issues suggests that factors intrinsic to maternal physiology may be interfering with lactation [51]. This was captured in the low milk production cluster, wherein low milk supply was nearly universal and occurred in relative isolation. In our study, endocrine, cardiovascular, and gastrointestinal conditions were associated with the low milk production cluster. Endocrine conditions are an established risk factor for impaired lactation [51], through mechanisms related to insulin resistance, breast hypoplasia, and reduced prolactin responsiveness [52]. Previous studies have found that low milk supply was associated with diabetes in pregnancy [53], and that fewer women with polycystic ovary syndrome (PCOS) breastfed to 6 months relative to women without PCOS (44.3% vs. 54.2%, respectively) [54]. Our findings related to cardiovascular and gastrointestinal conditions are novel and merit further investigation. Lactation interfaces with several body systems, stimulating changes in hormone activity, gastrointestinal blood flow, metabolic rate, and cardiac output [55, 56]. Yet the influence of underlying maternal disease pathophysiology on lactation physiology has received minimal attention to date. Alternatively, given that we could not verify self-reported low milk supply with objective measurements of milk volume, our findings may reflect a hypervigilance among mothers with these conditions towards milk adequacy as opposed to differences in actual milk production.

Strengths of this study include the prospective design, community-based sampling of diverse chronic conditions, and graded measurement of breastfeeding difficulties in all women who initiated breastfeeding regardless of duration. However, some limitations should be considered. Validity of self-reported chronic conditions is imperfect relative to clinical exams or medical records; however, a quarter of the sample were recruited directly from obstetric clinics specializing in chronic medical disorders or using mailed letters sent to women with diagnostic codes for chronic conditions and pregnancy. Of the remaining participants recruited through social media or other methods, over 80% reported receiving prenatal care from an obstetrician and/or specialist physician. It is therefore likely that most women recruited into the sample have a true clinical diagnosis. Chronic condition types were based on the ICD system, but often involved grouping heterogeneous conditions. For example, endocrine conditions included type 1 diabetes, an autoimmune disease, as well as PCOS, a metabolic gynecologic disorder. Additional research on individual conditions is needed to verify the associations we observed and investigate distinct underlying mechanisms. Our use of complete case analysis for handling missing covariate data (< 3% of observations) may have slightly reduced the precision of our estimates [57]. Finally, compared to the maternal population in Alberta, the MaCI sample slightly under-represented mothers who were younger than 24 years of age, who self-identified as BIPOC race/ethnicity, or who did not hold a post-secondary degree. Caution is needed when generalizing findings from the MaCI Study to these underrepresented groups.

Our findings have important clinical implications. Women should be counselled prenatally about the potential breastfeeding difficulties they may experience related to their chronic condition. Given that our data suggests half of mothers with chronic conditions who choose to breastfeed will experience low milk production or ineffective latch, health care providers should closely monitor breastfeeding experiences for these clusters of breastfeeding difficulties and promptly offer evidence-based interventions when they arise [17, 45]. For example, mothers reporting low milk supply in the absence of latch issues should be evaluated for potential underlying physiologic or psychosocial contributors and counselled on appropriate feeding frequency, infant weight gain, and wet diapers, as well as supported to feed or express more frequently or initiate galactagogues as indicated to increase supply [17, 45]. More broadly, future research that employs latent class analysis of breastfeeding difficulties would be valuable to ascertain whether the clusters we identified in mothers with chronic conditions are similarly observed in the general maternal population and to compare the distributions of each.

## Conclusion

In summary, we identified three clusters of breastfeeding difficulties in mothers with chronic conditions, corresponding to physiologically expected lactation changes, ineffective latch, and low milk production. Approximately half of mothers with chronic conditions belonged to either the ineffective latch or low milk production cluster. Relative to the physiologically expected cluster, endocrine, cardiovascular, and gastrointestinal conditions were associated with the low milk production cluster and gastrointestinal conditions were additionally associated with the ineffective latch cluster. These findings can aid with differentiating and treating breastfeeding difficulties in clinical practice and suggest that the influence of disease pathophysiology should be considered when providing early postpartum breastfeeding support.

## Supplementary Information

**Additional file 1:** **Supplementary Table 1.** Comparison of characteristics among mothers in the analytic sample, MaCI baseline cohort, and province of Alberta. **Supplementary Table 2.** Prevalence of moderate-to-severe breastfeeding difficulties experienced between birth and 6 weeks postpartum. **Supplementary Table 3.** Model fit indices from latent class analysis of breastfeeding difficulty clusters. **Supplementary Table 4. **Breastfeeding difficulty clusters experienced by women according to method of breast milk feeding used between birth and 6 weeks postpartum.

## Data Availability

The datasets used and/or analyzed in this study are available from the corresponding author, NVS, on reasonable request.

## Abbreviations

AIC: Akaike information criterion
BIC: Bayesian information criteria
BMI: Body mass index
CI: Confidence interval
EPDS: Edinburgh Postnatal Depression Scale
ICD: International Classification of Diseases
LCA: Latent class analysis
LICO: Low income cut-off
MaCI: Motherhood and Chronic Illness
RRR: Relative risk ratio
SABIC: Sample size-adjusted BIC
STAI: Spielberger State-Trait Anxiety Inventory

## References

[1] Victora C, Bahl R, Barros A, França G, Horton S, Krasevec J (2016). Breastfeeding in the 21st century: epidemiology, mechanisms, and lifelong effect. Lancet.

[2] Lodge CJ, Tan DJ, Lau M, Dai X, Tham R, Lowe AJ (2015). Breastfeeding and asthma and allergies: a systematic review and meta-analysis. Acta Paediatr.

[3] Nguyen B, Gale J, Nassar N, Bauman A, Joshy G, Ding D (2019). Breastfeeding and cardiovascular disease hospitalization and mortality in parous women: Evidence from a large Australian cohort study. J Am Heart Assoc.

[4] Stuebe AM, Schwarz EB, Grewen K, Rich-Edwards JW, Michels KB, Foster EM (2011). Duration of lactation and incidence of maternal hypertension: a longitudinal cohort study. Am J Epidemiol.

[5] Stuebe A, Rich-Edwards J, Willett W, Manson J, Michels K (2005). Duration of lactation and incidence of type 2 diabetes. JAMA.

[6] World Health Organization. Breastfeeding. http://www.who.int/nutrition/topics/exclusive_breastfeeding/en/.

[7] Scime NV, Patten SB, Tough SC, Chaput KH (2020). Maternal chronic disease and breastfeeding outcomes: a Canadian population-based study. J Matern Neonatal Med.

[8] Odom E, Li R, Scanlon K, Perrine C, Grummer-Strawn L (2013). Reasons for earlier than desired cessation of breastfeeding. Pediatrics.

[9] Li R, Fein SB, Chen J, Grummer-Strawn LM (2008). Why mothers stop breastfeeding: mothers’ self-reported reasons for stopping during the first year. Pediatrics.

[10] Brown CRL, Dodds L, Legge A, Bryanton J, Semenic S (2014). Factors influencing the reasons why mothers stop breastfeeding. Can J Public Heal.

[11] Rydström LL, Tavallali A, Sundborg E, Berlin A, Ranheim A (2021). Caught on the fringes of life: mothers’ lived experiences of initial breastfeeding complications. Qual Health Res.

[12] Palmér L, Carlsson G, Mollberg M, Nyström M (2012). Severe breastfeeding difficulties: Existential lostness as a mother—Women's lived experiences of initiating breastfeeding under severe difficulties. Int J Qual Stud Health Well-being..

[13] Williamson I, Leeming D, Lyttle S, Johnson S (2012). “It should be the most natural thing in the world”: exploring first-time mothers’ breastfeeding difficulties in the UK using audio-diaries and interviews. Matern Child Nutr.

[14] Brown A, Rance J, Bennett P (2016). Understanding the relationship between breastfeeding and postnatal depression: the role of pain and physical difficulties. J Adv Nurs.

[15] Chaput KH, Nettel-Aguirre A, Musto R, Adair CE, Tough SC (2016). Breastfeeding difficulties and supports and risk of postpartum depression in a cohort of women who have given birth in Calgary: a prospective cohort study. CMAJ Open.

[16] Brown A (2017). Breastfeeding as a public health responsibility: a review of the evidence. J Hum Nutr Diet.

[17] Committee on Obstetric Practice Breastfeeding Expert Work Group (2021). ACOG committee opinion #820: breastfeeding challenges. Obstet Gynecol.

[18] McDonald S, Pullenayegum E, Chapman B, Vera C, Giglia L, Fusch C (2012). Prevalence and predictors of exclusive breastfeeding at hospital discharge. Obstet Gynecol.

[19] Scime NV, Metcalfe A, Nettel-Aguirre A, Tough SC, Chaput KH (2021). Association of prenatal medical risk with breastfeeding outcomes up to 12 months in the all our families community-based birth cohort. Int Breastfeed J.

[20] Henninger ML, Irving SA, Kauffman TL, Naleway AL, Kurosky SK, Rompala K (2017). Predictors of breastfeeding initiation and maintenance in an integrated healthcare setting. J Hum Lact.

[21] Scime NV, Lee S, Jain M, Metcalfe A, Chaput K (2021). A scoping review of breastfeeding in women with chronic diseases. Breastfeed Med.

[22] Berg M, Erlandsson LK, Sparud-Lundin C (2012). Breastfeeding and its impact on daily life in women with type 1 diabetes during the first six months after childbirth: a prospective cohort study. Int Breastfeed J.

[23] Marasco L, Marmet C, Shell E (2000). Polycystic ovary syndrome: a connection to insufficient milk supply?. J Hum Lact.

[24] Stuebe A, Meltzer-Brody S, Pearson B, Pedersen C (2015). Maternal neuroendocrine serum levels in exclusively breastfeeding mothers. Breastfeed Med.

[25] Lipson JG, Rogers JG (2000). Pregnancy, birth, and disability: women’s health care experiences. Health Care Women Int.

[26] Schaefer K (2004). Breastfeeding in chronic illness: the voices of women with fibromyalgia. MCN Am J Matern Nurs.

[27] Powell RM, Mitra M, Smeltzer SC, Long-Bellil LM, Smith LD, Rosenthal E (2018). Breastfeeding among women with physical disabilities in the United States. J Hum Lact.

[28] Birru Talabi M, Eudy AM, Jayasundara M, Haroun T, Nowell WB, Curtis JR (2021). Tough choices: exploring medication decision-making during pregnancy and lactation among women with inflammatory arthritis. ACR Open Rheumatol.

[29] Williams D, Webber J, Pell B, Grant A, Sanders J, Choy E (2019). “Nobody knows, or seems to know how rheumatology and breastfeeding works”: women’s experiences of breastfeeding whilst managing a long-term limiting condition–a qualitative visual methods study. Midwifery.

[30] Agency for Healthcare Research & Quality. Beta Chronic Condition Indicator (CCI) for ICD-10-CM: Healthcare Cost and Utilization Project (HCUP). 2018. https://www.hcup-us.ahrq.gov/toolssoftware/chronic_icd10/chronic_icd10.jsp. 21413206

[31] Brown CRL, Dodds L, Attenborough R, Bryanton J, Rose AE, Flowerdew G (2013). Rates and determinants of exclusive breastfeeding in first 6 months among women in Nova Scotia: a population-based cohort study. CMAJ Open.

[32] Gianni ML, Bettinelli ME, Manfra P, Sorrentino G, Bezze E, Plevani L (2019). Breastfeeding difficulties and risk for early breastfeeding cessation. Nutrients.

[33] Kelleher CM (2006). The physical challenges of early breastfeeding. Soc Sci Med.

[34] Wambach KA (1997). Breastfeeding intention and outcome: a test of the theory of planned behavior. Res Nurs Heal.

[35] Lamontagne C, Hamelin AM, St-Pierre M (2008). The breastfeeding experience of women with major difficulties who use the services of a breastfeeding clinic: a descriptive study. Int Breastfeed J.

[36] Nelson AM (2012). A meta-synthesis related to infant feeding decision making. MCN Am J Matern Nurs.

[37] Hagenaars J, McCutcheon A (2002). Applied Latent Class Analysis.

[38] Laursen B, Hoff E (2006). Person-centered and variable-centered approaches to longitudinal data. Merrill Palmer Q.

[39] Nylund KL, Asparouhov T, Muthén BO (2008). Deciding on the number of classes in latent class analysis and growth mixture modeling: a Monte Carlo simulation study. Struct Equ Model.

[40] Cox JL, Holden JM, Sagovsky R (1987). Detection of postnatal depression: development of the 10-item Edinburgh postnatal depression scale. Br J Psychiatry.

[41] Gibson J, McKenzie-Mcharg K, Shakespeare J, Price J, Gray R (2009). A systematic review of studies validating the Edinburgh postnatal depression scale in antepartum and postpartum women. Acta Psychiatr Scand.

[42] Marteau TM, Bekker H (1992). The development of a six-item short-form of the state scale of the Spielberger State-Trait Anxiety Inventory (STAI). Br J Clin Psychol.

[43] Grant K, McMahon C, Austin M (2008). Maternal anxiety during the transition to parenthood: a prospective study. J Affect Disord.

[44] Cooklin AR, Amir LH, Nguyen CD, Buck ML, Cullinane M, Fisher JRW (2018). Physical health, breastfeeding problems and maternal mood in the early postpartum: a prospective cohort study. Arch Womens Ment Health.

[45] Rosen-Carole C, Stuebe AM, Lawrence R, Lawrence R (2022). Chapter 7: Practical Management of the Nursing “Dyad”. Breastfeeding: A Guide for the Medical Profession.

[46] Buck ML, Amir LH, Cullinane M, Donath SM (2014). Nipple pain, damage, and vasospasm in the first 8 weeks postpartum. Breastfeed Med.

[47] Amir LH, Baeza C, Charlamb JR, Jones W (2021). Identifying the cause of breast and nipple pain during lactation. BMJ.

[48] Neifert MR (2004). Breastmilk transfer: Positioning, latch-on, and screening for problems in milk transfer. Clin Obstet Gynecol.

[49] Woolf CJ (2011). Central sensitization: Implications for the diagnosis and treatment of pain. Pain.

[50] Amir LH, Jones LE, Buck ML (2015). Nipple pain associated with breastfeeding: Incorporating current neurophysiology into clinical reasoning. Aust Fam Physician.

[51] Farah E, Barger MK, Klima C, Rossman B, Hershberger P (2021). Impaired lactation: Review of delayed lactogenesis and insufficient lactation. J Midwifery Women’s Heal.

[52] Rassie K, Mousa A, Joham A, Teede HJ (2021). Metabolic conditions including obesity, diabetes, and polycystic ovary syndrome: Implications for breastfeeding and breastmilk composition. Semin Reprod Med.

[53] Riddle SW, Nommsen-Rivers LA (2016). A case control study of diabetes during pregnancy and low milk supply. Breastfeed Med.

[54] Joham AE, Nanayakkara N, Ranasinha S, Zoungas S, Boyle J, Harrison CL (2016). Obesity, polycystic ovary syndrome and breastfeeding: an observational study. Acta Obstet Gynecol Scand.

[55] Lawrence R, Lawrence R, Lawrence R (2022). Chapter 3: Physiology of Lactation. Breastfeeding: A Guide for the Medical Profession.

[56] Betts CB, Quackenbush A, Anderson W, Marshall NE, Schedin PJ (2020). Mucosal immunity and liver metabolism in the complex condition of lactation insufficiency. J Hum Lact.

[57] Pedersen AB, Mikkelsen EM, Cronin-Fenton D, Kristensen NR, Pham TM, Pedersen L (2017). Missing data and multiple imputation in clinical epidemiological research. Clin Epidemiol.

