# Supplementary material for: Breastfeeding difficulties in the first 6 weeks postpartum among mothers with chronic conditions: a latent class analysis
Source: BMC Pregnancy Childbirth. 2023 Feb 2;23:90. doi: 10.1186/s12884-023-05407-w (PMC9893695; doi:10.1186/s12884-023-05407-w)
Supplement: Supplementary file 1 — Additional file 1: Supplementary Table 1. Comparison of characteristics among mothers in the analytic sample, MaCI baseline cohort, and province of Alberta. Supplementary Table 2. Prevalence of moderate-to-severe breastfeeding difficulties experienced between birth and 6 weeks postpartum. Supplementary Table 3. Model fit indices from latent class analysis of breastfeeding difficulty clusters. Supplementary Table 4. Breastfeeding difficulty clusters experienced by women according to method of breast milk feeding used between birth and 6 weeks postpartum. [file 12884_2023_5407_MOESM1_ESM.docx]

**Supplementary Material**

Breastfeeding difficulties in the first 6 weeks postpartum among mothers with chronic conditions: A latent class analysis

**Supplementary Table 1**. Comparison of characteristics among mothers in the analytic sample, MaCI baseline cohort, and province of Alberta

**Supplementary Table 2.** Prevalence of moderate-to-severe breastfeeding difficulties experienced between birth and 6 weeks postpartum

**Supplementary Table 3.** Model fit indices from latent class analysis of breastfeeding difficulty clusters

**Supplementary Table 4.** Breastfeeding difficulty clusters experienced by women according to method of breast milk feeding used between birth and 6 weeks postpartum

**Supplementary Table 1**. Comparison of characteristics among mothers in the analytic sample, MaCI baseline cohort, and province of Alberta

| Characteristic | Analytic Sample | MaCI Baseline Cohort | Alberta  (2014-2018) |
| --- | --- | --- | --- |
|  | % | % | % |
| Number of participants | 348 | 371 | NA |
| Maternal age in years |  |  |  |
| 24 or younger | 4.0 | 4.6 | 15.0 |
| 25-34 | 73.6 | 73.6 | 65.8 |
| 35 or older | 22.4 | 21.9 | 19.2 |
| Race/ethnicity |  |  |  |
| White | 78.1 | 77.6 | 61.9 |
| Black, Indigenous, or Person of Colour | 21.9 | 22.4 | 38.1 |
| Household income |  |  |  |
| < $60,000 CAD | 23.9 | 24.6 | 24.5 |
| ≥ $60,000 CAD | 76.1 | 75.4 | 75.5 |
| Education |  |  |  |
| Less than post-secondary degree | 19.3 | 19.7 | 24.9 |
| Post-secondary degree | 80.7 | 80.3 | 75.1 |
| BMI |  |  |  |
| Mean in kg/m^2^ | 27.4 | 27.4 | 24.6 |
| Parity |  |  |  |
| Primiparous | 48.1 | 48.9 | 41.2 |
| Multiparous | 51.9 | 51.1 | 58.8 |

NA: not applicable; values reported come from multiple data sources.

Alberta: Scime et al. https://doi.org/10.1111/aogs.13769; Statistics Canada. Canadian Community Health Survey - Annual Component, 2017-2018. CCHS-82M0021X-E-2017-2018-Annual.

**Supplementary Table 2.** Prevalence of moderate-to-severe breastfeeding difficulties experienced between birth and 6 weeks postpartum

|  | Prevalence  % |
| --- | --- |
| Tired/fatigued | 68.4 |
| Leaking breasts | 44.0 |
| Sore nipples | 43.1 |
| Engorged breasts | 35.9 |
| Low milk supply | 32.5 |
| Latch problems | 30.7 |
| Baby fussy | 30.2 |
| Feeds too frequent | 27.9 |
| Baby sleepy | 27.0 |
| Difficulty positioning | 24.4 |
| Baby weight concerns | 24.4 |
| Cracked nipples | 18.4 |

**Supplementary Table 3.** Model fit indices from latent class analysis of breastfeeding difficulty clusters

|  | 1 Class | 2 Class | 3 Class | 4 Class |
| --- | --- | --- | --- | --- |
| AIC | 5084.1 | 4887.6 | 4845.7 | 4816.1 |
| BIC | 5130.4 | 4983.9 | 4992.1 | 5012.6 |
| Sample size-adjusted BIC | 5130.4 | 4907.8 | 4840 | 4784.4 |
| Log likelihood | -2530.1 | -2418.8 | -2384.9 | -2357.1 |

AIC: Akaike information criterion, BIC: Bayesian information criterion

**Supplementary Table 4.** Breastfeeding difficulty clusters experienced by women according to method of breast milk feeding used between birth and 6 weeks postpartum

|  | At the breast  (n = 88) | Expressed milk  (n = 21) | At the breast and expressed  (n = 239) |
| --- | --- | --- | --- |
|  | % | % | % |
| Cluster 1: Physiologically Expected | 69.3 | 33.3 | 45.6 |
| Cluster 2: Low milk production | 11.4 | 38.1 | 16.7 |
| Cluster 3: Ineffective latch | 19.3 | 28.6 | 37.7 |
